# Association of morphological markers of flower buds and anthers with the pollen developmental stage in *Vicia faba* L. (*Fabaceae*)

**DOI:** 10.3389/fpls.2026.1863348

**Published:** 2026-08-05

**Authors:** Wiktor Skrzypkowski, Ibrahim Moshood, Jose M. Seguí-Simarro, Agnieszka Kiełkowska

**Affiliations:** 1 Department of Plant Biology and Biotechnology, Faculty of Biotechnology and Horticulture, University of Agriculture (UAK), Krakow, Poland; 2 Cell Biology Group - Instituto para la Conservación y Mejora de la Agrodiversidad Valenciana (COMAV) Institute, Universitat Politècnica de València, Valencia, Spain

**Keywords:** anther morphometry, faba bean, floral bud size, legumes, microspore, pollen

## Abstract

The precise identification of microspore and pollen at the optimal developmental stages to be induced towards embryogenesis (vacuolated microspores and young pollen) is essential for induction of *in vitro* androgenesis in plants. Such identification is not always easy, and it is especially difficult in recalcitrant species such as *Vicia faba*. The present study evaluates the relationship between floral bud and anther morphometric parameters, and microspore/pollen developmental stages in *V. faba* using various methods including fluorescent staining, differential interference contrast microscopy, and morphometry. We measured flower bud and anther length and width, grouping them at different intervals, and performed a detailed microscopical and anatomical analysis of buds, anthers and microspores/pollen at different stages. Our results demonstrated that flower buds in *V. faba* exhibit complex and irregular morphologies, with considerable variation in both sepal length and shape. Furthermore, the determination of microspore and pollen developmental stages in this species is constrained by pronounced developmental asynchrony and strong genotype dependence. Although anther length measurements correlate closely with microspore and pollen developmental stages, their practical use can be challenging. Therefore, measuring flower bud length, while excluding sepals, remains the most practical criterion for routine applications. Combining this refined morphometric approach with microscopic validation appears to be the most effective strategy for improving the identification of flower buds containing microspores or pollen at developmental stages suitable for androgenesis induction in this recalcitrant legume species.

## Introduction

*Vicia faba* (also known as the faba bean, broad bean, fava bean, or horse bean) is a member of the *Fabaceae* family, which includes many crop species of high economic importance. Species belonging to this botanical family are cultivated both as food for humans and animals and as a valuable component in crop rotation due to their symbiotic relationships with nitrogen-fixing rhizobial bacteria, which contribute to soil enrichment and structural improvement. The high commercial value of legumes is primarily determined by the rich protein content of their seeds. Additionally, they contain significant amounts of complex carbohydrates and dietary fiber, as well as lipids, bioactive peptides, and polyphenols (Carbas et al., 2023; Poteraș et al., 2024). Legumes are also widely used as fodder crops. The high commercial importance of leguminous plants, including *V. faba*, creates a strong demand for breeding efforts aimed at developing new cultivars with increased resistance to diseases and pests, as well as reduced susceptibility to stress factors such as drought, excess water, or osmotic stress caused by soil salinity. In recent years, there has been a growing demand for varieties suitable for cultivation in extensive farming systems or organic farming, which forces the development of biotechnology-based breeding tools (Luby and Shaw, 2009; Schaart et al., 2016; Zolkin et al., 2023). As a result, there is a strong need for homozygous forms, which are an essential component of any breeding program in both self- and cross-pollinated crops. In conventional plant breeding, such forms are obtained through self-fertilization and selection, which often takes many generations. Plant biotechnology methods, such as *in vitro* techniques and haploidization procedures, hold great potential in this area. Haploidization significantly shortens the process of obtaining fully homozygous forms, known as doubled haploids (DHs), generated by induction of haploid plants development and subsequently doubling their genome (Żur et al., 2022; Kiełkowska and Kiszczak, 2023). In self-pollinated crops, the DH lines with valuable traits can directly represent a new variety. In cross-pollinated crops DH plants are used as a replacement for inbreeding for the production of parental lines for hybrid varieties (Kiełkowska and Kiszczak, 2023; Skrzypkowski and Kiełkowska, 2024). *V. faba* L. is a partially allogamous crop whose outcrossing rates (about 50%) present distinct challenges in traditional breeding. There are no hybrid cultivars expressing heterosis in *V. faba*, as their production requires pollination control based on, e.g., a stable CMS system, which is not available in this species (Maalouf et al., 2019). *V. faba* cultivars available on the market are typically line varieties or synthetic cultivars that outperform inbred lines due to the partially expressed heterosis. Line varieties are genetically uniform plant populations derived from self-pollinated or highly inbred crops, consisting of individuals that are nearly 100% homozygous. Synthetic cultivars are developed by selecting a limited number of superior inbred lines as components and by subsequently producing seed from a mixed stand of these lines. The implementation of doubled haploid (DH) technology significantly accelerates the breeding process by compressing multiple generations of conventional self-pollination needed to produce inbred lines into a single step. For line varieties, this technique permits instant homozygous line fixation, bypassing laborious inbreeding cycles and accelerating the time to market. For synthetic cultivars, DH lines serve as highly uniform parental components (Syn-0). This eliminates the genetic variation associated with incomplete inbreeding, allowing breeders to mitigate the negative effects of unequal paternal outcrossing success and more reliably forecast heterotic yield outcomes in subsequent generation (Brünjes and Link, 2021).

Haploid or DH plants can be produced in nearly 400 different species (Seguí-Simarro et al., 2021a) through various ways involving both male and female haploid cells reprogrammed towards embryogenesis (Seguí-Simarro et al., 2021b; Murovec and Bohanec, 2011; Żur et al., 2022). Androgenesis involves the induction of embryogenesis from haploid cells of male origin (Seguí-Simarro, 2010). When male gametophyte precursors (microspores) are used, the process is known as microspore embryogenesis, which can be induced through two approaches: anther cultures, where whole anthers are isolated from flower buds and serve as the explant in culture, and isolated microspore cultures, a more laborious approach where microspores are released from anthers and cultured in liquid media (Seguí-Simarro, 2016). Despite differences in these techniques, they share a common crucial factor: the precise selection of the appropriate developmental stage of the male gametophyte precursor. This is essential for successfully reprogramming the developmental pathway of the microspore from the gametophytic to the sporophytic route, which produces a haploid or DH regenerant. In many plant species it is possible to distinguish several consecutive stages of microspore and pollen development, beginning with pollen mother cells (PMCs) and tetrads, followed by the uninucleate microspore stage, which can be subdivided into young, mid, and late vacuolated microspores. This is followed by the binucleate pollen stage, often further divided into early and late phases, and, in many species, by the trinucleate pollen stage resulting from the second mitotic division (Liberatore et al., 2019; Nguyen et al., 2022; Pagalla, 2023). In contrast, in *Vicia faba* such a detailed subdivision of the uninucleate stage is difficult to achieve. Following tetrad dissolution, only two distinct stages can be reliably distinguished: young microspores and late vacuolated microspores characterized by a polarized nucleus. Likewise, the stages following the first pollen mitosis are limited to young binucleate pollen and mature pollen grains, as the trinucleate pollen stage is not observed in anthers in this species (Skrzypkowski et al., 2023).

There is a broad consensus in the literature regarding the narrow developmental window during microspore/pollen development that is suitable for induction of *in vitro* androgenesis. This window in the majority of species revolves around the stages of late vacuolated microspores and early bicellular pollen (Parra-Vega et al., 2013; Liberatore et al., 2019; Salas et al., 2012; Seguí-Simarro et al., 2021b). Indeed, reports on induction using previous or later stages are rare and typically describe severe induction treatments (Binarova et al., 1997; Seguí-Simarro and Nuez, 2005; Perera et al., 2014). In legumes (i.e. *Lens culinaris*, *Lupinus* sp., *Cicer arietinum, Pisum sativum*, *Lathyrus sativus*, and *V. faba*), the determination of the suitable stages is still an open topic where more information is needed. While some studies have described the inducible stages in legumes as those between the mid-unicellular and early bicellular stages (Khan and Ghosh, 1983; Croser et al., 2004; Ochatt et al., 2009; Skrzypkowski and Kiełkowska, 2024), other studies reported induction without specifying the developmental stage used (reviewed in Skrzypkowski and Kiełkowska, 2024). In particular, *V. faba* has been described as recalcitrant to haploidization, which explains why progress in developing effective haploidization methods and procedures has been minimal (Skrzypkowski and Kiełkowska, 2024). The few available reports on haploidization in *V. faba* have focused on androgenesis induced mostly from microspores and typically resulted in callus formation (Paratasilpin, 1978; Hesemann, 1980; Shlahi et al., 2012; Küçükrecep and Tekdal, 2022). However, the ploidy level of the calli produced was rarely reported, and plant regeneration was not achieved (Skrzypkowski and Kiełkowska, 2024).

Since the efficiency of androgenesis induction strongly depends on the accurate determination of the right microspore/pollen developmental stages, it is crucial to find ways to correlate such stages with easily identifiable morphological markers in the explants containing these stages, i.e. entire flower buds or excised anthers. Here, we present a study that correlates measurable morphological parameters of *V. faba* flower buds and anthers with the developmental stages of microspores and pollen. The aim is to identify an easily applicable morphological marker to enable an accurate and repeatable determination of the microspore/pollen developmental stages suitable for androgenesis induction in *V. faba*.

## Materials and methods

### Plant material and growth conditions

**Figure 1:**
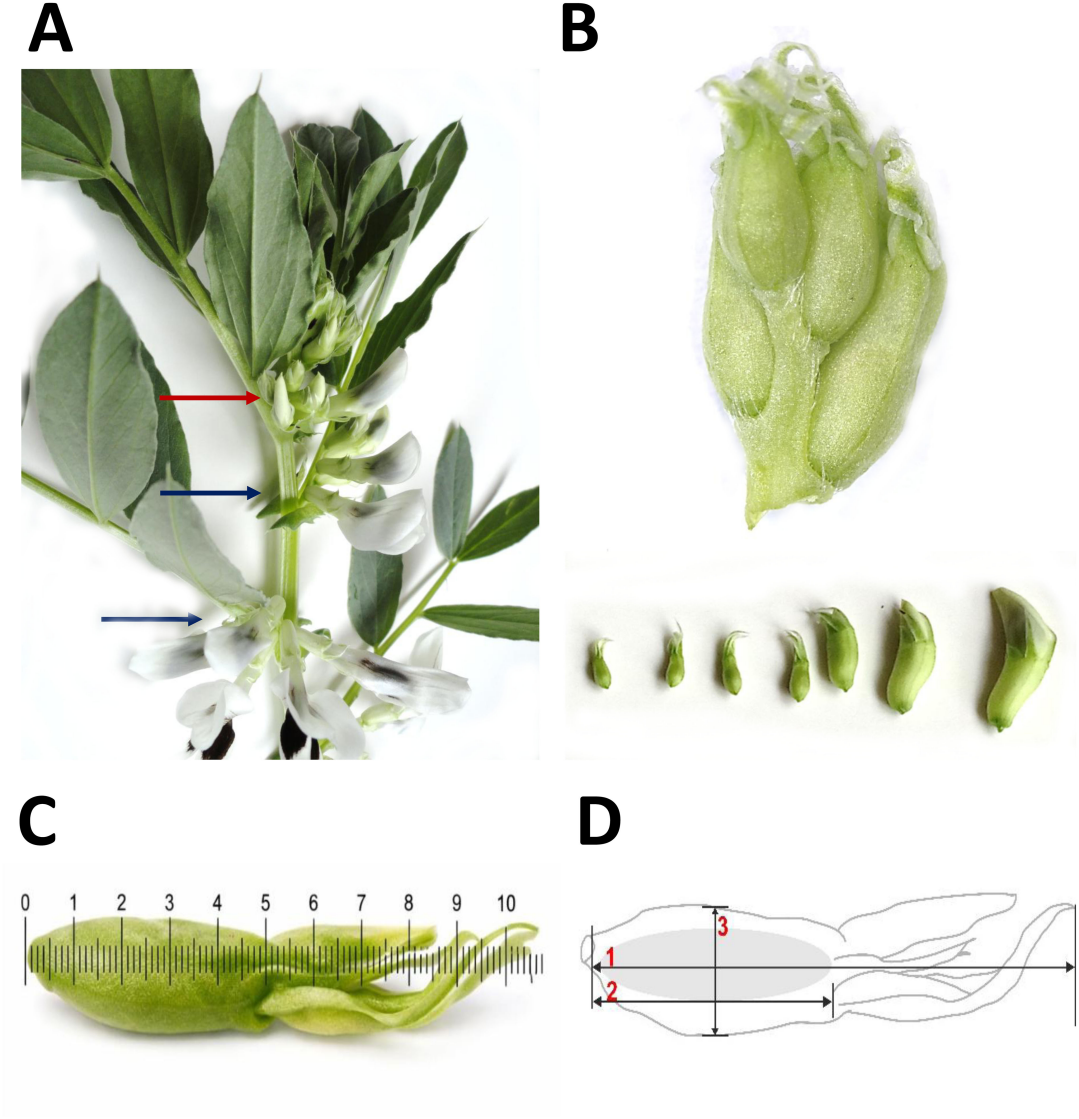
Inflorescence of *Vicia faba* L. and the measurement methodology for flower bud dimensions. **(A)** fragment of a stem with infloresceces at various stages of development (marked with arrows) produced in leaf axils. Blue arrows mark inflorescences with open flowers; the red arrow marks an inflorescence with floral buds. **(B)** close-up of a single inflorescence containing floral buds at various stages of development. **(C)** flower bud observed under a stereomicroscope equipped with a micrometric eyepiece (in mm). **(D)** schematic representation of the measurement protocol: (1) bud length measured including the sepal tip; (2) bud length measured without sepal tips; and (3) bud width measured at the widest point of the flower bud.

Four commercially available *Vicia faba* cultivars (‘Bartek’, ‘Bolero’ and ‘Bonus’ from PlantiCo, Zielonki. Sp. z.o.o, Poland, and ‘White Windsor’ from PNOS Ożarów Mazowiecki, Poland) were investigated. All tested plant materials were line varieties. The experiments were performed in the facilities of the Faculty of Biotechnology and Horticulture, UAK, Krakow, Poland. Seeds of all cultivars were sown in 12 L pots containing a 1:1 mixture of compost soil (Aura; Hollas Group, Pasłęk, Poland) and peat substrate (Hartmann Universal; Hartmann Polska, Poznań, Poland). For each cultivar, four pots with 4–5 plants per pot in three replications were prepared. The experiment was conducted in completely randomized design. The donor plants were cultivated in a growth chamber under controlled conditions, with a 16/8-hour photoperiod provided by sodium lamps (Lucalox LU600W/PSL, GE Lighting, Budapest, Hungary). The temperature was maintained at 18–20 °C during the day and 15–16 °C at night. The plants received optimal irrigation and fertilization throughout the growth period. The donor plants entered the flowering phase approximately two months after seed sowing. *V. faba* plants produced several consecutive inflorescences on each plant. The inflorescences grew from the leaf axils (**Figure 1A**), and each consisted of several (typically 4-8) flower buds (**Figure 1B**). For the analyses, whole inflorescences, including floral buds at different stages of maturation (**Figure 1B**), were collected from all nodes, excluding opened flowers (**Figure 1A** blue arrows), which contained bicellular pollen exclusively (Skrzypkowski and Kiełkowska, 2024). Inflorescences were collected in the morning and immediately transported to the laboratory, where they were placed in a vessel on moist paper to prevent dehydration. The plant material was analysed on the day of collection.

### Measurement procedures of *V. faba* flower buds

Measurements were performed using a Leica CLS 50 X stereoscopic microscope (Leica Microsystems, Wetzlar, Germany) equipped with a micrometric eyepiece (**Figure 1C**). The floral bud length and width and the length and width of the excised anthers from each bud were measured and recorded. Measurements of flower bud length and width were conducted following the scheme illustrated in **Figure 1D**. Bud length was measured using two methods: (1) from the receptacle to the sepal tip, or (2) from the receptacle to the base of sepal separation, depending on the experimental setup. Bud width (3) was measured at the widest part of the bud, approximately at ¾ of the length. After measurements, each floral bud was secured, tagged and subjected to anther measurements, anatomical analyses, and analyses of microspore/pollen developmental stages.

### Experimental design

Three separate experiments were performed using buds at various developmental stages, ranging from approximately 3 to 10 mm.

The first experiment was conducted to roughly identify the approximate bud length ranges corresponding to specific microspore or pollen developmental stages. For this, ‘Bonus’, ‘Bolero’ and ‘Bartek’ were used to measure and sort bud lengths at 1 mm intervals using method 1 (**Figure 1D**). This experiment comprised eight size bins (e.g. 3mm, 4 mm etc.), with 10 buds analysed per cultivar for each bin.

In the second experiment, samples from the same three cultivars as in first experiment were sorted at narrower bud length intervals of 0.5 mm (e.g., 4.0–4.4 mm, 4.5–4.9 mm, etc.) using method 1 (**Figure 1D**), resulting in fifteen distinct size bins. For each length bin, 3 to 6 buds per cultivar were analysed. The same buds were subsequently used to measure bud width, as well as anther length and width. Regarding bud width, samples per cultivar were categorized into eleven size bins based on 0.1 mm intervals (e.g., 1.2 mm, 1.3 mm, etc.). For anther metrics per cultivar, five size bins were established for length (e.g., 0.45–0.59 mm, 0.60–0.74 mm, etc.) and four for width (e.g., 0.35–0.49 mm, 0.50–0.64 mm, etc.). A single *V. faba* bud contains 10 anthers, and for anther metrics all anthers from minimum 3 buds in each size bins was analysed.

The third experiment was carried out to evaluate to what extent sepal length distorts the correlation between flower bud length and the corresponding developmental stage. Each bud length was measured with 0.1 mm precision, both including and excluding sepals (methods 1 and 2 in **Figures 1D**, respectively) in cultivars ‘Bonus’ and ‘White Windsor’. Bud length including sepals was categorized into fourteen size bins (e.g., 3.0–3.4 mm, 3.5–3.9 mm, etc.), whereas bud length excluding sepals was divided into eight size bins (e.g., 1.5–1.9 mm, 2.0–2.4 mm, etc.).

### Anatomical analysis of anthers

For anatomical analysis, *V. faba* flower buds at various developmental stages (ranging from approximately 3 to 10 mm) were collected and fixed in Carnoy’s solution (absolute ethanol + glacial acetic acid 3:1 v/v). Fixation was performed for 48 h in a refrigerator at 4 ± 1 °C. Following fixation, samples were dehydrated in a graded ethanol series (70%, 80%, 90% *v/v*, 15* min* each) and left overnight in absolute ethanol. Then, buds were immersed and embedded in Technovit^®^ 7100 resin (Kulzer, Hanau, Germany) as described in Kiełkowska and Dziurka, 2021. Polymerized resin blocks containing plant samples were then sectioned into 4 µm slices using a Leica RM2145 rotary microtome (Leica Microsystems, Wetzlar, Germany) fitted with a carbide blade (Leica TC-65, KAWA.SKA, Zalesie Górne, Poland). Sections were stained with 1% (*w/v*) toluidine blue (Sigma-Aldrich, Darmstadt, Germany) and mounted with Entellan™ (Merck, Darmstadt, Germany). Microscopic observations were subsequently conducted using an Axio Imager M2 microscope (Carl Zeiss, Göttingen, Germany) in bright field mode. For each analysed bud length intervals two floral buds were embedded, sliced and analysed.

### DAPI staining and DIC microscopy

DAPI (4′,6-diamidino-2-phenylindole) staining was carried out for a rapid assessment of microspore/pollen developmental stages in collected flower buds. Buds of *V. faba* ranging from 3 to 10 mm in length were selected, and their anthers were carefully dissected. Isolated anthers were macerated directly on a glass slide in a drop of DAPI staining solution (2.5 µg mL⁻¹ DAPI, 7.7 mM Tris-HCl, 10 mM spermine tetrahydrochloride, 10 mM NaCl, 2.2% (v/v) hexylene glycol, and 0.25% (v/v) Triton™ X-100), then mixed in equal parts with glycerol as described in Skrzypkowski et al., 2023. The DAPI solution was stored in a dark glass bottle at 4 °C to maintain stability. Following maceration, any remaining anther tissue was gently removed before placing a coverslip over the sample. Fluorescence microscopy was performed using an Axio Imager M2 (Carl Zeiss, Göttingen, Germany) equipped with the appropriate DAPI filter set (Zeiss filter set 02; λEx = 365 nm, λEm > 420 nm). A minimum of 100 cells per slide were counted, with one slide prepared per bud and minimum 3 buds analysed for each bud size bin (covering bud/anther length and width) and cultivar. The percentage of cells in each stage from the total population of observed cells was calculated.

Differential interference contrast (DIC) observations were performed using the same microscope slides that were previously used for fluorescence observations with DAPI staining. DIC imaging was carried out using an Axio Imager M2 microscope (Carl Zeiss, Göttingen, Germany) equipped with appropriate DIC optics, including Nomarski prisms and a dedicated DIC condenser. This technique was used to validate DAPI observations, and it was particularly helpful in proper identification of pollen development at the earliest stages i.e. pollen mother cells and tetrads. Here, a minimum of 50 cells per slide were counted, with one slide prepared per bud and minimum 3 buds analysed for each bud size bin and cultivar.

### Statistical analysis

The mean values for each factor and standard error of the means (SEM) were calculated. The overall effect of the factor was determined using a multifactor analysis of variance (ANOVA) and mean separation was done using Tukey’s honestly significant difference (HSD) test at a significance level of 0.05. Statistical analysis was performed using Statistica software (TIBCO Software Inc., Palo Alto, CA, USA). The regression coefficients and ANOVA for regression and Pearson’s correlation were calculated and graphically represented using Microsoft Excel 365 (Microsoft Corporation, Redmond, USA). The statistical analysis results for all charts have been included in the **Supplementary Materials**.

## Results

### Morphological and cytological analysis of anthers

**Figure 2:**
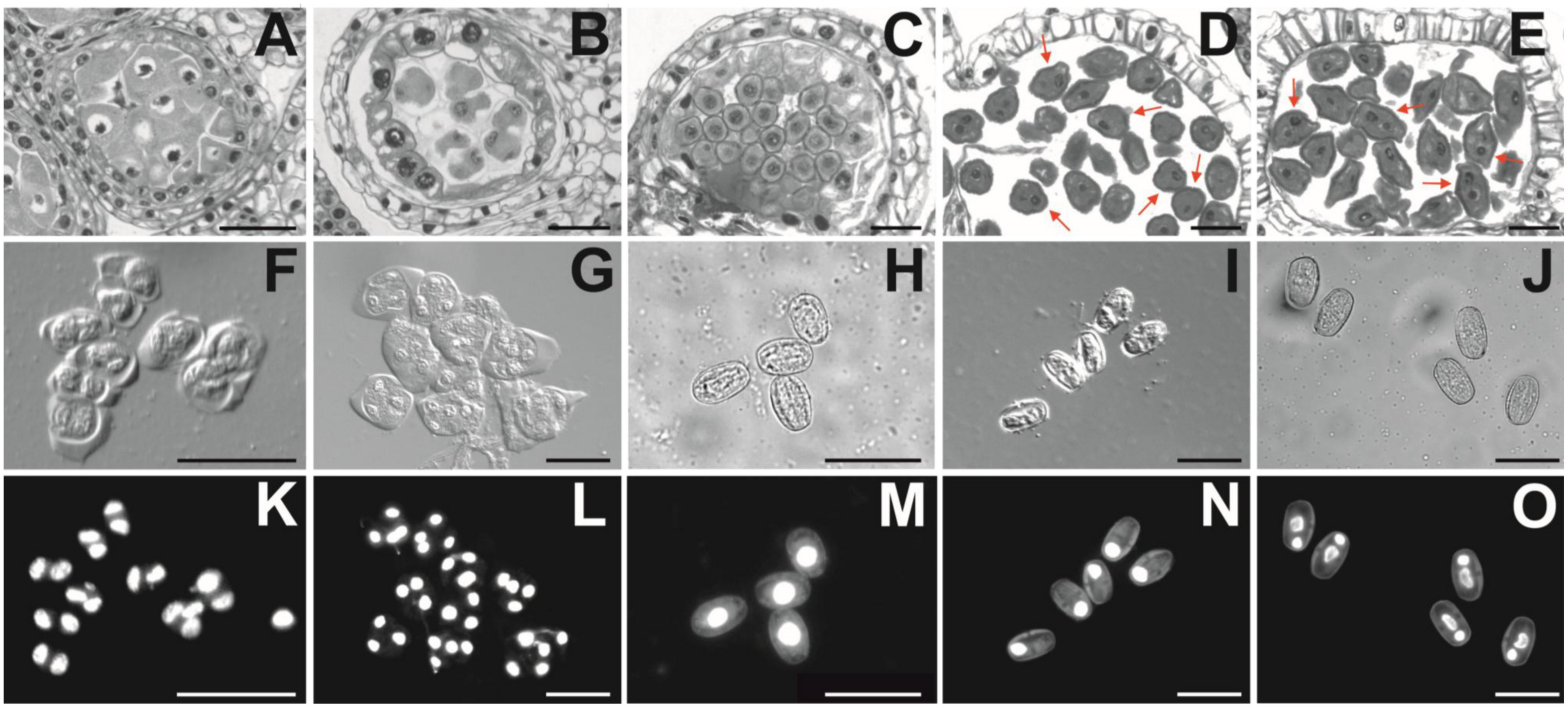
Morphological and cytological characterization of anthers within floral buds of *Vicia faba* differing in length. Top row shows cross sections of anthers within Technovit-embedded flower buds stained with toluidine blue; the mid and bottom rows show DIC microscopy (mid) and fluorescence images (bottom) of DAPI-stained cells extracted from anthers. Observed stages are as follows: pollen mother cells (PMC) **(A, F, K)**; tetrads **(B, G, L)**; young microspores **(C, H, M)**; vacuolated microspores (arrows in **D, I, N**); early pollen (arrows in **E, J, O**). Scale bar: 50 μm.

Analysis was conducted on ‘Bonus’ flower buds to preliminarily screen the different developmental stages: pollen mother cells (PMCs), tetrads, young microspores (with centrally located nuclei), vacuolated microspores (with polarized nuclei), and early (binucleated) pollen (**Figure 2**). These developmental stages in *V. faba* can be readily distinguished using markers such as cell shape, nucleus number, position and morphology, and the thickness of the callose wall of PMCs and tetrads. No trinucleate pollen grains were observed.

### Experiment 1

**Figure 3:**
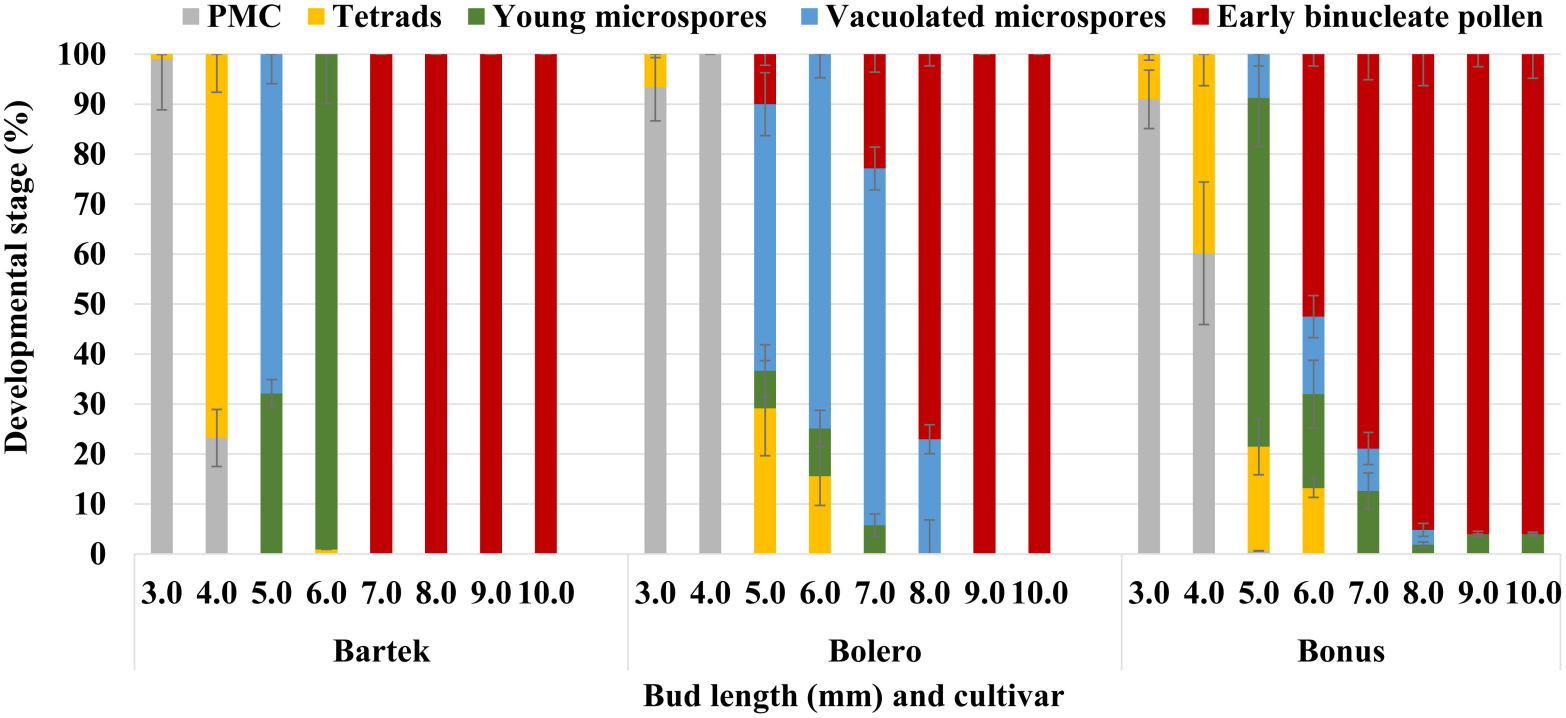
Distribution of developmental stages of microspores and pollen in *V. faba* depending on flower bud length (intervals 1 mm) and cultivar. Bars are means ± SEM. Statistical analysis results are available in the **Supplementary Materials**.

To preliminarily assess the association between floral bud morphology and microspore/pollen developmental stage, anthers were dissected from buds 3–10 mm in length (1 mm interval; **Figure 3**; **Supplementary Materials**). We observed differences in the developmental stage distribution depending on genotype and bud length. In the smallest buds (3–4 mm), PMCs predominated in ‘Bonus’ and ‘Bolero’ (60–100%), whereas ‘Bartek’ showed a lower proportion of PMCs and a predominance of tetrads (~77% at 4 mm). Young microspores were present at 5–6 mm buds in all cultivars, reaching up to 99% in ‘Bartek’ (and ca. 6–7% in ‘Bonus’ and ‘Bolero’). Vacuolated microspores were abundant in the intermediate buds (5–8 mm), dominating in ‘Bolero’ (53–75%) but remaining minor in ‘Bonus’ (3–16%) and limited to a single bud class (5 mm) in ‘Bartek’ (~68%). Early pollen was observed in larger buds, becoming the predominant or sole stage in the largest buds of all cultivars, with 100% representation in ‘Bartek’ at 7–10 mm, 52–96% in ‘Bonus’ and 77–100% in ‘Bolero’ at comparable bud lengths. These results highlight both a clear genotype-specific progression of microspore/pollen development and the co-occurrence of multiple stages within individual bud length bins.

### Experiment 2

**Figure 4:**
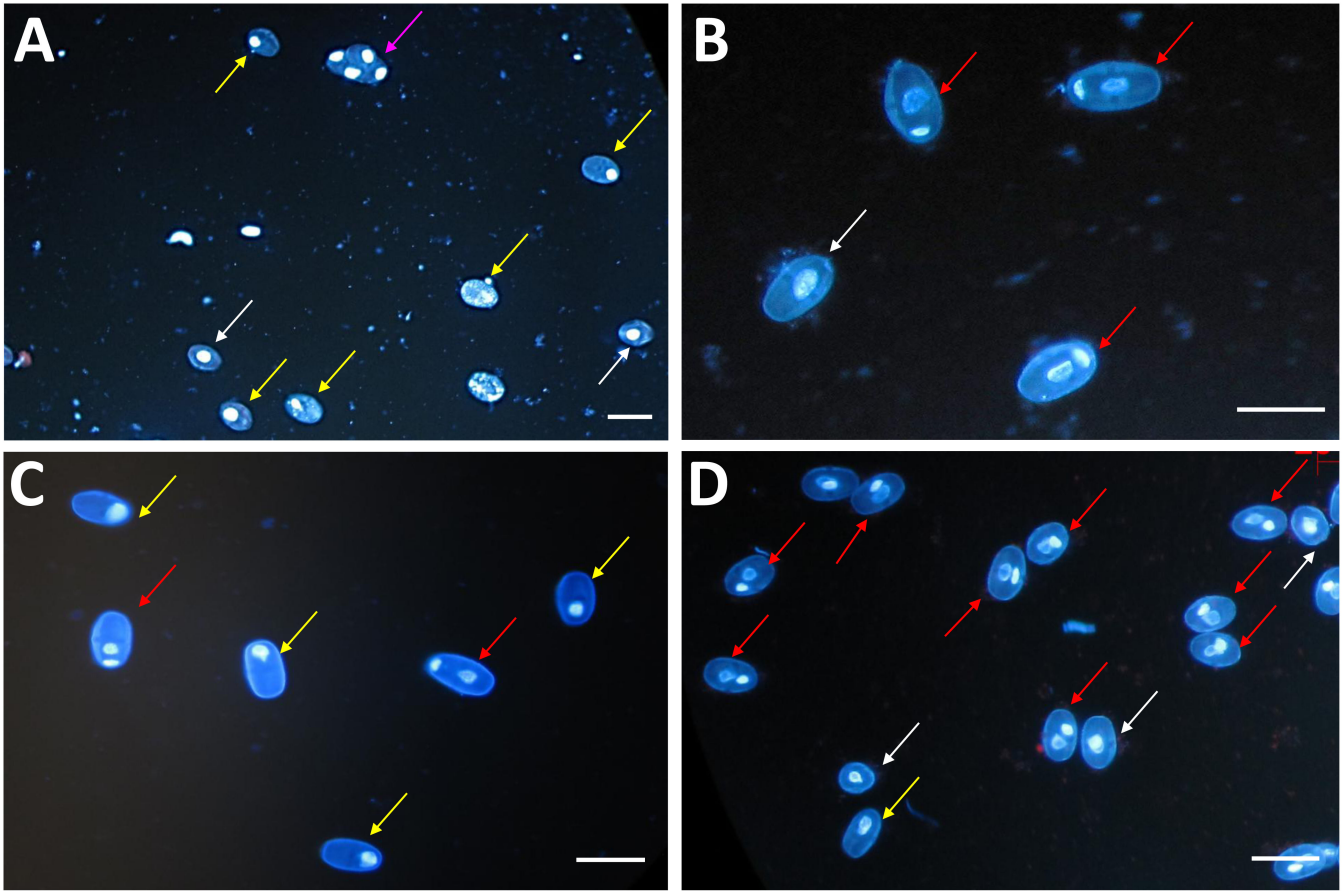
Representative fluorescence microphotographs of cells in anthers of *V.faba* stained with DAPI showing showing the coexistence of different developmental stages within the same anthers. **(A)** Tetrad (pink arrow), young microspores (white arrows) and vacuolated microspores (yellow arrows). **(B)** Young microspore (white arrows) together with early binucleate pollen grains (red arrow). **(C)** Vacuolated microspores (yellow arrows) and early binucleate pollen grains (red arrow). **(D)** Young microspores (white arrows), vacuolated microspore (yellow arrow) together with early binucleate pollen grains (red arrows). Scale bar: 20 μm.

**Figure 5:**
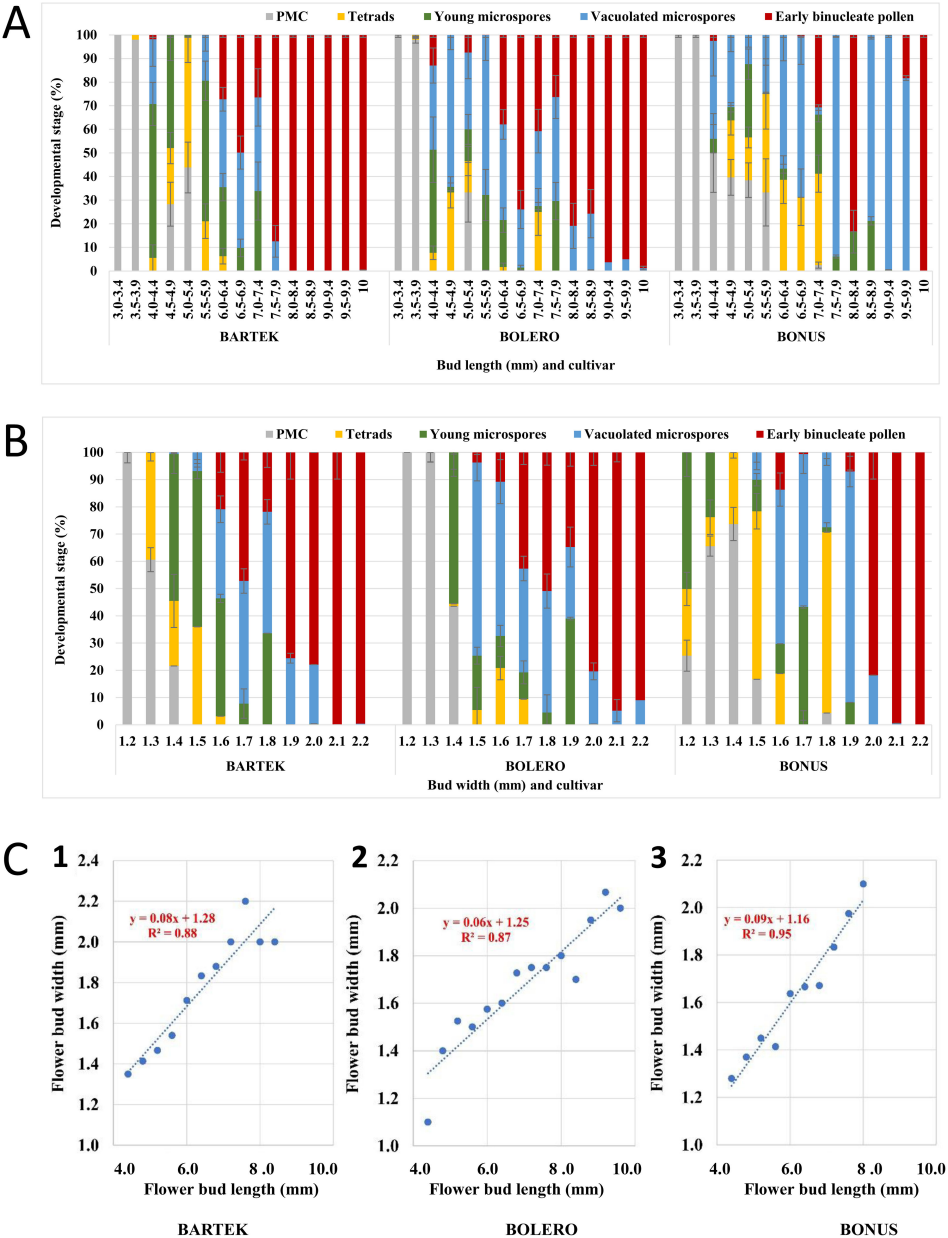
Relations of microspore and pollen developmental stages from bud morphology in three *V. faba* cultivars. Percentages of microspores and pollen developmental stages in dependency on: **(A)** flower bud lengths (0.5 mm intervals), **(B)** - flower bud widths (0.1 mm intervals). Bars are means ± SEM. **(C)** Scatter plots of flower bud length and width in *V. faba* cultivars ‘Bartek’ (1), ‘Bolero’ (2) and ‘Bonus’ (3). The blue line represents the linear regression; y - regression formula, R^2^ - coefficient of determination. The regression was statistically significant according to ANOVA (p < 0.05). Correlation coefficients **(r)** were +0.94 for ‘Bartek’, +0.94 for ‘Bolero’ and +0.96 for ‘Bonus’. Statistical analysis results for all above plots are available in the **Supplementary Materials**.

Based on the overlap of developmental stages observed in Experiment 1, bud length was measured with higher precision (0.5 mm intervals) and then grouped into size bins (e.g., 3–3.4 mm, 3.5–3.9 and further up to 10 mm, in total fifteen bins). This binning approach allowed for a more systematic presentation of the results and enabled clearer associations between morphological traits and microspore/pollen developmental stages. All tested parameters, including bud length and width and anther length and width, were recorded for the same samples. Two and occasionally three different developmental stages coexisted in the same flower bud (**Figure 4**), which contributed to variability within a given flower bud length and/or width (**Figures 5A, B**).

### Floral bud morphometry

DAPI analysis of developmental stage distribution relative to bud length and width (**Figures 5A, B**; **Supplementary File S1**) did not provide a clear or consistent picture. Buds of the same length, as in Experiment 1, also contained a mixture of cells at different developmental stages across all cultivars. The most consistent and coherent pattern was found in ‘Bartek’, where bud length and width closely corresponded to successive pollen stages. Early stages dominated in the smallest buds, followed by a gradual transition through intermediate forms, with advanced stages (vacuolated microspores and early binucleate pollen) clearly prevailing in larger buds. Importantly, intermediate stages disappeared in a logical sequence, and the largest buds contained almost exclusively pollen.

In contrast, ‘Bolero’ exhibited less stable pattern. Although the same sequence of developmental stages was present, multiple stages frequently co-occurred across a wide range of bud size bins. For example, in anthers of 6.0–6.4 mm buds, vacuolated microspores and early bicellular pollen were dominant, but accompanied by tetrads (1.7%) and young microspores (19.9%). While there was a visible shift from early to advanced stages—particularly an increase in early binucleate pollen accompanied by a decline in vacuolated microspores—the frequencies of intermediate stages fluctuated considerably, making it difficult to assign precise developmental phases to specific bud dimensions.

The least consistent pattern was observed in ‘Bonus’, where pollen development appeared irregular, with absence or reappearance of intermediate stages between adjacent size bins. Early stages such as PMCs and tetrads persisted across unexpectedly broad size ranges and even reappeared at later stages. Intermediate forms were sometimes absent and then detected again in larger buds, while transitions to advanced stages were not linear. In some cases, developmental progression seemed to reverse, with vacuolated microspores dominating again in the largest buds instead of early binucleate pollen. For example, young microspores were entirely absent in the anthers of 6.5–6.9 mm buds, and in anthers of larger buds (7.4–8.9) they were detected again. In anthers of 9.0–9.4 mm buds, only vacuolated microspores were observed, while in the anthers of 9.5–9.9 mm buds, they (81.7%) co-occurred with early binucleate pollen. The latter stage was the sole developmental form present in the anthers of the longest buds (10 mm). This lack of continuity indicates a poor correlation between bud length and developmental stage. In summary, neither bud length nor width are reliable markers to predict microspore/pollen developmental stages in *V. faba*, as they are limited by the lack of correlation between measurements and stages.

A strong positive correlation between bud length and width was observed in all three analysed cultivars (**Figure 5C**; **Supplementary Materials**). Linear regression models revealed that an increase in bud length was consistently associated with an increase in mean bud width. ‘Bonus’, Bolero’ and ‘Bartek’, exhibited high coefficients of determination (R^2^: 0.95, 0.87 and 0.88, respectively), indicating a strong linear association and minimal scatter of data points around the fitted regression line. Regression slopes indicated a fast width increase per length unit, with 0.09 for ‘Bonus’, 0.08 for ‘Bartek’ and 0.06 for ‘Bolero’. These results highlight a reliable and cultivar-consistent relationship between bud length and width, which may serve as a practical reference when selecting buds for developmental stage assessments for *in vitro* androgenesis experiments.

### Anther morphometry

**Figure 6:**
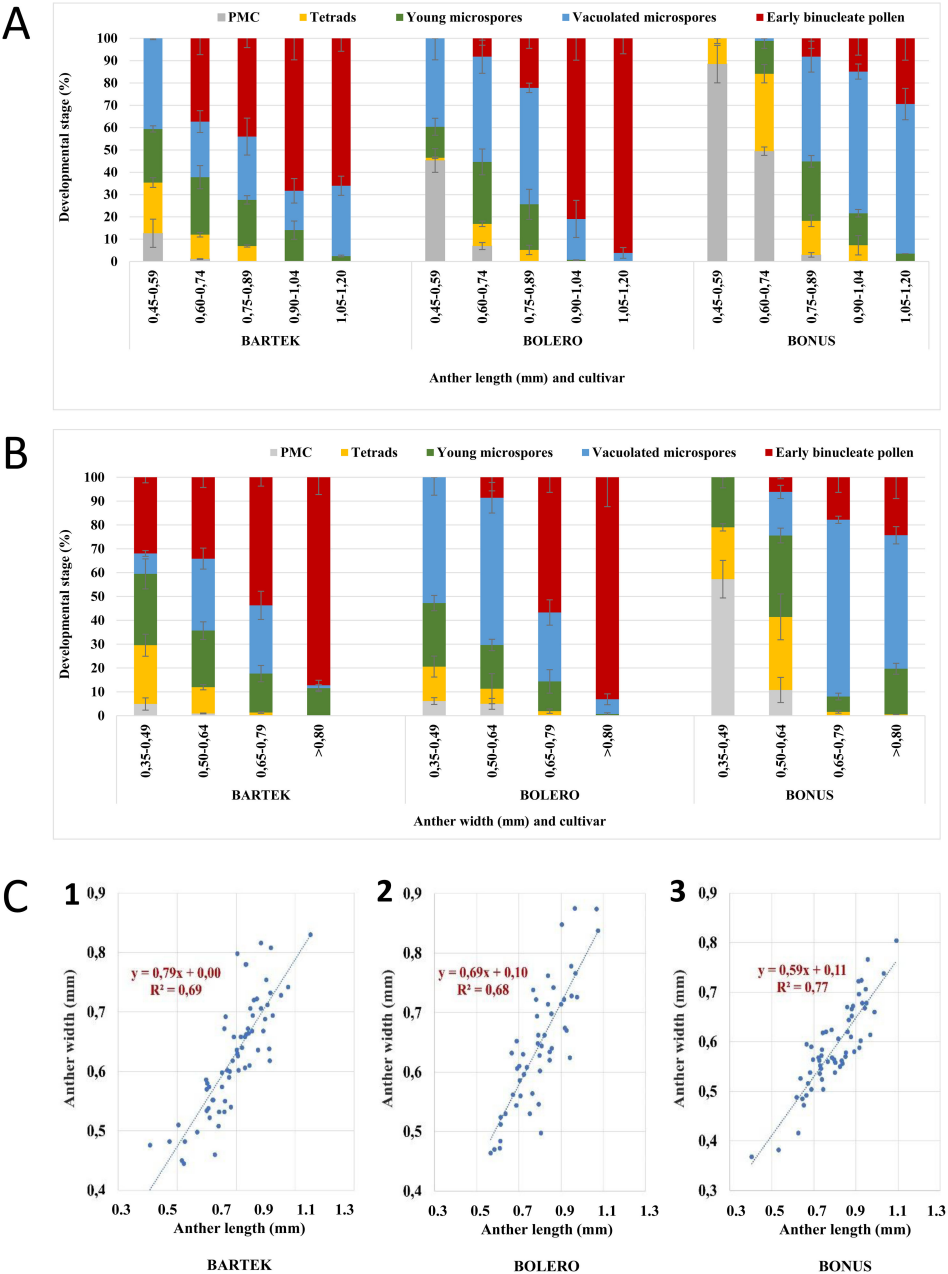
Relations of microspore and pollen developmental stages from anther morphology in three *V. faba* cultivars. Percentages of microspores and pollen developmental stages for different: **(A)** anthers length, **(B)** anthers width. Bars are means ± SEM. **(C)** Scatter plots of anther length and width in *V. faba* cultivars ‘Bartek’ (1), ‘Bolero’ (2), and ‘Bonus’ (3). The blue line represents the linear regression; y - regression formula, R^2^ - coefficient of determination. The regression was statistically significant according to ANOVA (p < 0.05). Correlation coefficients **(r)** were +0.84 for ‘Bartek’, +0.83 for ‘Bolero’ and +0.88 for ‘Bonus’. Statistical analysis results for all above plots are available in the **Supplementary Materials**.

We also evaluated the correlation between microspore/pollen developmental stages and anther length and width. As to anther length (**Figure 6A**; **Supplementary Materials**), results consistently showed different stages coexisting in anthers of the same length, and a gradual succession of developmental stages in progressively larger anthers. In smaller buds (0.45–0.59) of ‘Bartek’ and ‘Bolero’ a mixture of four early stages of pollen development was observed. ‘Bonus’ was more coherent as two stages were detected in those buds. A clear majority of vacuolated microspores and early pollen grains were found in 0.9–1.2 mm buds for all tested cultivars. Anther width also showed a coherent and consistent distribution of microspore and pollen developmental stages (**Figure 6B**; **Supplementary Materials**). In this case, the anther width range where a clear majority of vacuolated microspores and early pollen grains were found in 0.6 mm and larger for all cultivars. Although there were also inconsistencies i.e. ‘Bartek’, where young pollens were found even in the smallest buds. In summary, anther length and width are more reliable parameters than flower bud length and width to estimate the different microspore and pollen developmental stages contained within them.

A significant positive correlation between anther length and width was observed in all tested cultivars (**Figure 6C**; **Supplementary Materials**). ‘Bonus’ exhibited the highest coefficient of determination (R^2^= 0.7694), indicating a strong linear association and relatively low scatter of data points around the fitted regression line. ‘Bolero’ and ‘Bartek’ showed slightly lower, yet still substantial, coefficients of determination (R^2^= 0.6809 and 0.6904, respectively), reflecting moderately strong correlations with somewhat greater variability in the data. These results highlight a consistent and cultivar-specific correlation between anther length and width, which may be useful for selecting anthers at specific developmental stages.

### Experiment 3

**Figure 7:**
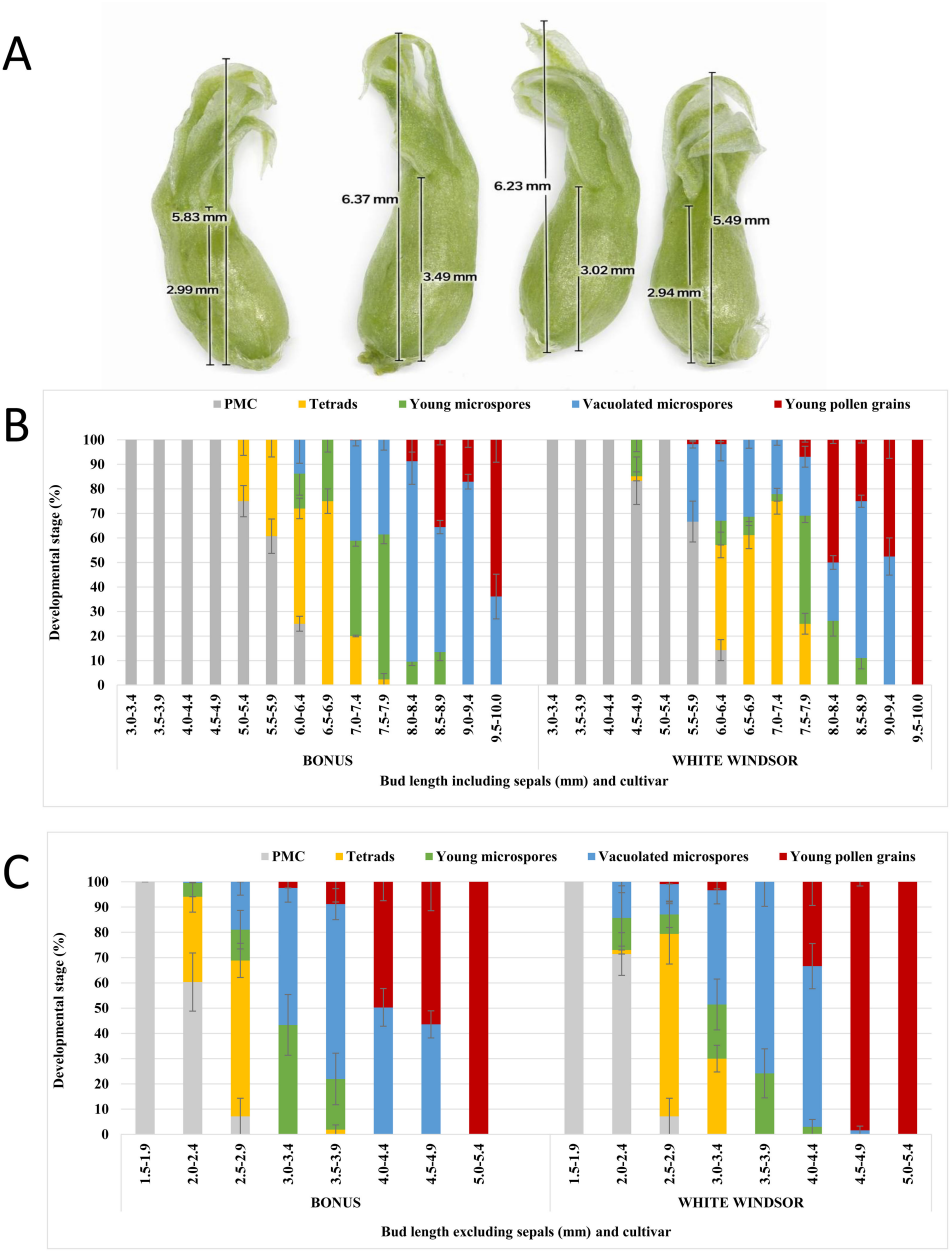
The impact of flower bud anatomy on the accuracy of pollen developmental stage analysis in two cultivars of *V. faba.* **(A)** representative flower buds of ‘Bonus’ illustrating their irregular shapes and uneven growth of sepals. Sepals often curl and are not uniformly long, making difficult accurate and reproducible bud length measurements when sepals are included. Percentages of microspores and pollen developmental stages in different flower bud lengths measured including sepals **(B)** and excluding sepals **(C)** in ‘Bonus’ and ‘White Windsor’. Bars are means ± SEM. Statistical analysis results for all above plots are available in the **Supplementary Materials**.

In the previous experiments we noticed that *V. faba* sepals consistently exhibit irregular growth, curling and uneven shapes (**Figure 7A**; **Supplementary Materials**), which could increase variability in measurements and therefore affect the accuracy of microspore/pollen developmental stage assessment. Thus, we next measured flower bud lengths in ‘Bonus’ and ‘White Windsor’ (1) including and (2) excluding sepals as described in Materials and Methods. To facilitate graphical representation, lengths were grouped in 0.5 mm intervals. The latter cultivar was introduced to validate results obtained in two previous experiments.

When sepals were included, slight inconsistencies in stage distributions were observed (**Figure 7B**; **Supplementary Materials**), as expected base on Experiments 1 and 2. In ‘Bonus’, development was relatively continuous but strongly overlapping. PMCs were observed across a wide range of bud lengths (6 bins, 3–6.4 mm), while tetrads, young microspores, and vacuolated microspores co-occurred in buds of 5 and 7.9 mm in length. Early binucleate pollen appeared only in larger buds (8–10 mm) and increased gradually. In ‘White Windsor’, the pattern was more discontinuous and irregular. Although early stages declined with bud length and advanced stages eventually dominated, several gaps were observed—most notably the absence of tetrads and young microspores in 5–5.9 mm buds and the temporary disappearance of early binucleate pollen in 6.5–7.4 mm buds, while there were observed in smaller buds having 5.5–6.4 mm.

As opposed to this, excluding sepals from measurements reduced variability and produced clearer and more coherent developmental patterns, with a progressive replacement of younger by older developmental stages as bud length increases (**Figure 7C**). In both cultivars, early stages (PMCs) dominated in the smallest buds (1.5–2.4 mm) and gradually declined with increasing bud length, while tetrads and young microspores appeared in intermediate bud size bins (2.5–4.9 mm). Vacuolated microspores spanned a relatively wide bud length range (2.5–4.9 mm). At the same time, early binucleate pollen increased with bud length, marking advancement toward later developmental stages.

In summary, *V. faba* floral bud anatomy affects the accuracy of microspore/pollen developmental stage assessment. Including sepals in bud length measurements increases variability due to their irregular and uneven growth and shape. Instead, exclusion of sepals provides more consistent developmental trends and reduces the irregularities caused by sepal variability, which significantly improves the precision for microspore/pollen stage assessment.

## Discussion

Recalcitrance to haploidization remains a major limitation for DH production in many important crop species such as tomato, cassava, or the whole *Fabaceae* family (Croser et al., 2006; Seguí-Simarro, 2016; Perera et al., 2014; Skrzypkowski and Kiełkowska, 2024). To the best our knowledge, no *Fabaceae* breeding program uses DHs on a routine basis so far. In all of the inducible species, the precise identification of the microspore and pollen developmental stage competent for reprogramming is a prerequisite for successful induction of haploidization (Seguí-Simarro et al., 2021b; Hale et al., 2022). Studies performed in some legumes, including *Pisum sativum* (Ochatt et al., 2009), *Lens culinaris* (Deswal, 2018; Vijaipandurangam, 2019; Gupta et al., 2020), *Lupinus* spp. (Skrzypek et al., 2008; Simioniuc et al., 2010; Kozak et al., 2012), and *Cicer arietinum* (Huda et al., 2001), are in line with the broad consensus around the vacuolated microspore and early pollen stages as the most responsive. To identify these stages, different rapid, non-destructive methods based on the study of morphological markers such as the calyx-bud ratio, or anther or flower bud length, width and/or flower bud or anther pigmentation, have been successfully used in other species (Kiełkowska et al., 2018; Liberatore et al., 2019; Nguyen et al., 2022; Parra-Vega et al., 2013; Salas et al., 2012; Żur et al., 2022). Unfortunately, this does not apply to *V. faba*, where accurately predicting microspore and pollen developmental stages based on floral bud characteristics is significantly more challenging (Skrzypkowski et al., 2023).

We performed a series of experiments to identify the major limitations affecting precise developmental staging of floral buds in *V. faba*, and found three major limitations: (1) asynchrony of microspore/pollen development in different anthers, (2) large differences among genotypes, and (3) large variability in sepal size and shape. The first major constraint is the pronounced developmental asynchrony of microspores/pollen within floral buds. The stages of microsporogenesis and microgametogenesis we observed in *V. faba*, including PMCs, tetrads, young microspores, vacuolated microspores, early pollen, and even mature bicellular pollen, correspond well with the classical sequence of angiosperm microspore/pollen development (Liu and Wang, 2021). Interestingly, no trinucleate pollen grains were observed in our study, confirming that in *V. faba* pollen is released at the bicellular stage during anthesis. Our observation aligns with findings reported for many other legume species (Teixeira et al., 2002; Jiang et al., 2019a, Jiang et al., 2019b; Skrzypkowski et al., 2023) as well as with the general pattern observed in the majority of angiosperms (Williams et al., 2014).

We showed that multiple developmental stages frequently coexisted in different buds of the same size, in the same bud and even within individual anthers. A similar scenario has also been described in *Pisum sativum* (Li et al., 2024). This partial developmental overlap would be characteristic of legumes, and even more pronounced in *V. faba*, significantly complicating the selection of donor materials. The reasons behind observed of pollen asynchrony in *V. faba* has not been characterized, but data in other species showed that it might have different causes. In general, the development is synchronized until microsporocyte meiosis I (Mamun et al., 2005); nevertheless, meiosis II can be asynchronous (Ekici, 2014). The asynchronous events might also involve, microspore vacuolization (Shim et al., 2009), followed by new cytoplasm formation and later reduction of vacuoles (Zhang et al., 2002) and non-simultaneous pollen mitosis across all cells (Lora et al., 2009). It has been reported that environmental stress (such as extreme temperatures or drought) may cause developmental failures and partial or total pollen impairment (Song et al., 2015).

Second, we showed that the correlation between microspore/pollen developmental stages and flower bud parameters is strongly dependent on the genotype. The cultivars ‘Bartek’, ‘Bolero’, and ‘Bonus’ differed markedly in the distribution and proportions of each stage across flower bud length and width intervals. For example, although developmental stage overlap was common in intermediate bud lengths, the bud length bins where anthers contain only vacuolated microspores and early bicellular pollen were narrower in ‘Bartek’ (7.5–7.9 mm; **Figure 5**) than in the other cultivars. This cultivar also exhibited the most coherent relationship between bud width and microspore/pollen progression. In contrast, ‘Bonus’ showed a less uniform and more discontinuous developmental pattern, including the persistence of PMCs and tetrads in relatively large buds, and abrupt transitions between developmental stages. ‘Bolero’ displayed the most gradual progression, with an extensive overlap of developmental stages across broad floral bud size bins. These differences were also reflected in the regression slopes, pointing out the strong genotype dependence of microspore/pollen developmental stages. Thus, while floral bud width could serve as a useful proxy in selected cultivars, microscopic validation remains essential for the accurate identification of buds with microspores/pollen at the suitable stages for embryogenesis induction in each *V. faba* cultivar. In other words, bud length or width alone should not be regarded as a precise proxy for staging in *V. faba*. We also showed that anther length and width correlate with floral bud parameters and allows for an identification of microspore/pollen progression, from PMCs to early pollen, more reliably than floral bud measurements alone, as also observed in other species (Adhikari and Kang, 2017; Marchant and Walbot, 2022). Nevertheless, the practical application of anther measurements in this species appears limited. Measurements must be performed immediately after dissection, prior to culture initiation, which increases the risk of tissue desiccation and contamination. Therefore, despite its correlation with microspore/pollen progression, anther morphometry would be less suitable for routine staging.

Third, *V. faba* floral buds are uniformly green and anthers are yellowish-green across developmental stages. No consistent external changes in morphology can be reliably associated to internal developmental transitions. Furthermore, Experiments 1 and 2 revealed the complex, irregular and curved morphology of sepals. To check out to what extent sepal morphology was behind the weak correlations found in these experiments, we designed Experiment 3 to compare measurements considering and excluding sepals. Exclusion of sepals significantly improved the correspondence of microspore/pollen developmental stage with bud length, allowing for a narrower delimitation of bud length ranges mostly containing vacuolated microspores and early pollen. Among all the approaches tested, bud length measurement excluding sepals coupled with DAPI testing provided the most practical compromise between developmental precision and technical feasibility.

## Conclusions

Determination of the optimal morphological markers for selection of the most suitable explants for induction of microspore embryogenesis in *V. faba* is challenging due to a number of limiting factors including (1) asynchrony of microspore/pollen development in different anthers, (2) large differences among genotypes, and (3) large variability in sepal size and shape. This study represents the first comprehensive experimental assessment of multiple factors influencing the accurate prediction of microspore/pollen developmental stages in *V. faba*, offering practical solutions to reduce the inconsistencies derived from these limitations. The most optimal approaches appear to be either bud length measurement excluding sepals coupled with meticulous analysis of developmental stages of microspore and pollen development or, alternatively, direct anther measurement; the latter, however, is more laborious and riskier, due to the possibility of explant dehydration or contamination prior to culture. Consequently, the study achieved its objective of identifying an easily applicable morphological marker enabling accurate and reproducible determination of developmental stages suitable for androgenesis induction in *V. faba*.

## Data Availability

The raw data supporting the conclusions of this article will be made available by the authors, without undue reservation.

## References

[B1] Adhikari P. B. Kang W. H. (2017). Association of floral bud and anther size with microspore developmental stage in Campari tomato. Hortic. Sci. Technol. 35, 608–617. doi: 10.12972/kjhst.20170065

[B2] Binarova P. Hause G. Cenklova V. Cordewener J. H. G. van Lookeren-Campagne M. M. (1997). A short severe heat shock is required to induce embryogenesis in late bicellular pollen of Brassica napus L. Sex Plant Reprod. 10, 200–208. doi: 10.1007/s004970050088 30311153

[B3] Brünjes L. Link W. (2021). Paternal outcrossing success differs among faba bean genotypes and impacts breeding of synthetic cultivars. Theor. Appl. Genet. 134, 2411–2427. doi: 10.1007/s00122-021-03832-z 33961063 PMC8277637

[B4] Carbas B. MaChado N. Pathania S. Brites C. Rosa E. A. Barros A. I. (2023). Potential of legumes: Nutritional value, bioactive properties, innovative food products, and application of eco-friendly tools for their assessment. Food. Rev. Intl 39, 160–188. doi: 10.1080/87559129.2021.1901292 37339054

[B5] Croser J. S. Lulsdorf M. Cheng B. Allen K. Wilson J. Dament T. . (2004). “ Embryogenesis from isolated microspores of chickpea and field pea - Progress towards a doubled haploid protocol as a tool for crop improvement”, in: Proceedings of the 4th International Crop Science Congress. (Brisbane, Australia: Agronomy Australia Proceedings), 255–256.

[B6] Croser J. S. Lülsdorf M. M. Davies P. A. Clarke H. J. Bayliss K. L. Mallikarjuna N. . (2006). Toward doubled haploid production in the Fabaceae: Progress, constraints, and opportunities. Crit. Rev. Plant Sci. 25, 139–157. doi: 10.1080/07352680600563850 37339054

[B7] Deswal K. (2018). Progress and opportunities in double haploid production in lentil (Lens culinaris Medik.), soybean (Glycine max L. Merr.) and chickpea (Cicer arietinum L.). J. Pharmac Phytochem 7, 3105–3109.

[B8] Ekici N. (2014). Microsporogenesis, pollen mitosis and pollen tube growth in Gagea villosa (Liliaceae). Biologia 69, 1323–1330. doi: 10.2478/s11756-014-0439-8

[B9] Gupta D. Dady R. H. Sambasivam P. Bar I. Azad M. Beera N. . (2020). “ Conventional and biotechnological approaches for targeted trait improvement in lentil,” in Accelerated Plant Breeding, vol. 3 . Eds. Gosal S. S. Wani S. H. ( Springer, Cham, Switzerland), 67–107. doi: 10.1007/978-3-030-47306-8_3

[B10] Hale B. Ferrie A. Chellamma S. Samuel J. P. Phillips G. C. (2022). Androgenesis-based doubled haploidy: Past, present, and future perspectives. Front. Plant Sci. 12, 751230. doi: 10.3389/fpls.2021.751230 35069615 PMC8777211

[B11] Hesemann C. U. (1980). Haploid cells in calli from anther culture of Vicia faba. Z Pflanzenzuecht 84, 23–29.

[B12] Huda S. Islam R. Bari M. A. Asaduzzaman M. (2001). Anther culture of chickpea. Int. Chickpea Pigeonpea Newsl. 8, 24–26.

[B13] Jiang Y. Davis A. R. Vujanovic V. Bueckert R. A. (2019b). Reproductive development response to high daytime temperature in field pea. J. Agron. Crop Sci. 205, 324–333. doi: 10.1111/jac.12328 40046247

[B14] Jiang Y. Lahlali R. Karunakaran C. Warkentin T. D. Davis A. R. Bueckert R. A. (2019a). Pollen, ovules, and pollination in pea: Success, failure, and resilience in heat. Plant Cell Environ. 42, 354–372. doi: 10.1111/pce.13427 30136298

[B15] Khan S. K. Ghosh P. D. (1983). *In vitro* induction of androgenesis and organogenesis in Cicer arietinum L. Curr. Sci. 52, 891–893.

[B16] Kiełkowska A. Adamus A. Baranski R. (2018). Haploid and doubled haploid plant production in carrot using induced parthenogenesis and ovule excision *in vitro*. Plant Cell. Culture Protoc. Methods Mol. Biol. 1815, 301–315. doi: 10.1007/978-1-4939-8594-4_21 29981131

[B17] Kiełkowska A. Dziurka M. (2021). Changes in polyamine pattern mediates sex differentiation and unisexual flower development in monoecious cucumber (Cucumis sativus L.). Physiol. Plantarum 171, 48–65. doi: 10.1111/ppl.13197 32840866

[B18] Kiełkowska A. Kiszczak W. (2023). History and current status of haploidization in carrot (Daucus carota L.). Agronomy 13, 676. doi: 10.3390/agronomy13030676 30654563

[B19] Kozak K. Galek R. Waheed M. T. Sawicka-Sienkiewicz E. (2012). Anther culture of Lupinus angustifolius: Callus formation and the development of multicellular and embryo-like structures. Plant Growth Regul. 66, 145–153. doi: 10.1007/s10725-011-9638-2 30311153

[B20] Küçükrecep A. Tekdal D. (2022). Microsporogenesis in faba bean (Vicia faba L.) grown in Mersin, Turkey. Plant Introduct 95/96, 68–74. doi: 10.46341/PI2022017

[B21] Li Z. Tang Y. Chen S. Yang Y. Zhang Y. Xiao L. . (2024). “ The correlation between the developmental stages of pea microspores and the morphology of flower buds”, in: BIO Web of Conferences (France: EDP Sciences), 142. 02008. doi: 10.1051/bioconf/202414202008

[B22] Liberatore C. M. Calabuig-Serna A. Rodolfi M. Chiancone B. Seguí-Simarro J. M. (2019). Phenological phases of flowering in hop (Humulus lupulus L.) and their correspondence with microsporogenesis and microgametogenesis. Sci. Hortic. 256, 108639. doi: 10.1016/j.scienta.2019.108639 38826717

[B23] Liu L. Wang T. (2021). Male gametophyte development in flowering plants: A story of quarantine and sacrifice. J. Plant Physiol. 258, 153365. doi: 10.1016/j.jplph.2021.153365 33548696

[B24] Lora J. Herrero M. Hormaza J. I. (2009). The coexistence of bicellular and tricellular pollen in Annona cherimola (Annonaceae): Implications for pollen evolution. Am. J. Bot. 96, 802–808. doi: 10.3732/ajb.0800167 21628235

[B25] Luby J. J. Shaw D. V. (2009). Plant breeders' perspectives on improving yield and quality traits in horticultural food crops. HortScience 44, 20–22. doi: 10.21273/HORTSCI.44.1.20

[B26] Maalouf F. Hu J. O’Sullivan D. M. Zong X. Hamwieh A. Kumar S. . (2019). Breeding and genomics status in faba bean (Vicia faba). Plant Breed. 138, 465–473. doi: 10.1111/pbr.12644 38826717

[B27] Mamun E. A. Cantrill L. C. Overall R. L. Sutton B. G. (2005). Cellular organisation in meiotic and early post-meiotic rice anthers. Cell Biol. Int. 29, 903–913. doi: 10.1016/j.cellbi.2005.08.001 16198129

[B28] Marchant D. B. Walbot V. (2022). Anther development - the long road to making pollen. Plant Cell 34, 4677–4695. doi: 10.1093/plcell/koac287 36135809 PMC9709990

[B29] Murovec J. Bohanec B. (2011). “ Haploids and doubled haploids in plant breeding,” in Plant Breeding. Ed. Abdurakhmonov I. Y. ( InTech, Rijeka, Croatia), 87–106. doi: 10.5772/29982

[B30] Nguyen M. L. Huyen T. N. B. T. Trinh D. M. Voronina A. V. (2022). Association of bud and anther morphology with developmental stages of the male gametophyte of melon (Cucumis melo L.). Vavilov J. Genet. Breed. 26, 146. doi: 10.18699/VJGB-22-18 35434488 PMC8983305

[B31] Ochatt S. Pech C. Grewal R. Conreux C. Lulsdorf M. Jacas L. (2009). Abiotic stress enhances androgenesis from isolated microspores of some legume species (Fabaceae). J. Plant Physiol. 166, 1314–1328. doi: 10.1016/j.jplph.2009.01.011 19324457

[B32] Pagalla D. B. (2023). Stages of microspore development in eggplant (Solanum melongena L.). Bioeduscience 7, 68–72. doi: 10.22236/jbes/7111357 40443743

[B33] Paratasilpin T. (1978). Vegetative development of field bean pollen grain cultured *in vitro*. J. Sci. Soc Thai 4, 139–145.

[B34] Parra-Vega V. González-García B. Seguí-Simarro J. M. (2013). Morphological markers to correlate bud and anther development with microsporogenesis and microgametogenesis in pepper (Capsicum annuum L.). Acta Physiol. Plantarum 35, 627–633. doi: 10.1007/s11738-012-1104-x 30311153

[B35] Perera P. I. Ordoñez C. A. Dedicova B. Ortega P. E. M. (2014). Reprogramming of cassava (Manihot esculenta) microspores towards sporophytic development. AoB Plants 6, plu022. doi: 10.1093/aobpla/plu022 24887001 PMC4061485

[B36] Poteraș C. B. Culețu A. Manolache F. A. (2024). Nutritional importance of lentil, lupin, chickpea and soy legumes: A review. Bull. Univ. Agric. Sci. Vet. Med. Cluj-Napoca Food. Sci. Technol. 81, 1–20. doi: 10.15835/buasvmcn-fst:2024.0007 42346774

[B37] Salas P. Rivas-Sendra A. Prohens J. Seguí-Simarro J. M. (2012). Influence of the stage for anther excision and heterostyly in embryogenesis induction from eggplant anther cultures. Euphytica 184, 235–250. doi: 10.1007/s10681-011-0569-9 30311153

[B38] Schaart J. G. van de Wiel C. C. Lotz L. A. Smulders M. J. (2016). Opportunities for products of new plant breeding techniques. Trends Plant Sci. 21, 438–449. doi: 10.1016/j.tplants.2015.11.006 26654659

[B39] Seguí-Simarro J. M. (2010). Androgenesis revisited. Botanical Rev. 76, 377–404. doi: 10.1007/s12229-010-9056-6 30311153

[B40] Seguí-Simarro J. M. (2016). “ Androgenesis in solanaceae,” in *In Vitro* Embryogenesis. Eds. Germanà M. A. Lambardi M. ( Springer Science+Business Media, New York, USA), 209–244. doi: 10.1007/978-1-4939-3061-6_9

[B41] Seguí-Simarro J. M. Belinchón Moreno J. Guillot Fernández M. Mir R. (2021a). “ Species with haploid or doubled haploid protocols,” in Doubled Haploid Technology. Ed. Seguí-Simarro J. M. ( Springer Science+Business Media, LLC, New York, USA), 41–103. doi: 10.1007/978-1-0716-1315-3_3 34270025

[B42] Seguí-Simarro J. M. Jacquier N. M. A. Widiez T. (2021b). “ Overview of *in vitro* and *in vivo* doubled haploid technologies,” in Doubled Haploid Technology. Ed. Seguí-Simarro J. M. ( Springer Science+Business Media, LLC, New York, USA), 3–22. doi: 10.1007/978-1-0716-1315-3_1 34270023

[B43] Seguí-Simarro J. M. Nuez F. (2005). Meiotic metaphase I to telophase II is the most responsive stage of microspore development for induction of androgenesis in tomato (Solanum lycopersicum). Acta Physiol. Plantarum 27, 675–685. doi: 10.1007/s11738-005-0071-x 30311153

[B44] Shim Y. S. Pauls K. P. Kasha K. J. (2009). Transformation of isolated barley (Hordeum vulgare L.) microspores: I. The influence of pretreatments and osmotic treatment on the time of DNA synthesis. Genome 52, 166–174. doi: 10.1139/g08-112 19234565

[B45] Shlahi S. A. Majeed D. M. Ismail E. N. (2012). Effect of the developmental stage of microspores, growth regulator and medium type on callus indication from broad bean Vicia faba anthers culture. J. Biot Res. 6, 81–90. doi: 10.24126/jobrc.2012.6.2.231

[B46] Simioniuc D. Burlacu-Arsene M. C. Morariu A. Lipșa F. D. (2010). Induction of the embryogenesis process in anther and microspores cultures at the Lupinus albus species. Lucrări Științifice, Universitatea de Stiinte Agricole Și Medicină Veterinară “Ion Ionescu de la Brad” Iași, Seria. Agronomie 53, 60–63.

[B47] Skrzypek E. Czyczyło-Mysza I. Marcińska I. Wędzony M. (2008). Prospects of androgenetic induction in Lupinus spp. Plant Cell. Tiss Organ Cult. 94, 131–137. doi: 10.1007/s11240-008-9396-7 30311153

[B48] Skrzypkowski W. Galek R. Adamus A. Kiełkowska A. (2023). Pollen development and stainability in Vicia faba L. and Lupinus angustifolius L. Agriculture 13, 2065. doi: 10.3390/agriculture13112065 30654563

[B49] Skrzypkowski W. Kiełkowska A. (2024). Current status of haploidization in cool-season grain legume crop species. Agriculture 14, 1031. doi: 10.3390/agriculture14071031 30654563

[B50] Song G. Wang M. Zeng B. Zhang J. Jiang C. Hu Q. . (2015). Anther response to high-temperature stress during development and pollen thermotolerance heterosis as revealed by pollen tube growth and *in vitro* pollen vigor analysis in upland cotton. Planta 241, 1271–1285. doi: 10.1007/s00425-015-2259-7 25672505

[B51] Teixeira S. P. Forni-Martins E. R. Ranga N. T. (2002). Development and cytology of pollen in Dahlstedtia Malme (Leguminosae: Papilionoideae). Bot. J. Linn. Soc 138, 461–471. doi: 10.1046/J.1095-8339.2002.00031.X 37945311

[B52] Vijaipandurangam K. D. (2019). Microspore culture effects on double haploid production in lentil (Lens culinaris Medik.). Int. J. Agric. Sci. Res. 8, 147–152.

[B53] Williams J. H. Taylor M. L. O'Meara B. C. (2014). Repeated evolution of tricellular (and bicellular) pollen. Am. J. Bot. 101, 559–571. doi: 10.3732/ajb.1300423 24663667

[B54] Zhang C. Guinel F. C. Moffat B. A. (2002). A comparative ultrastructural study of pollen development in Arabidopsis thaliana ecotype Columbia and male-sterile mutant apt1-3. Protoplasma 219, 59–71. doi: 10.1007/s007090200006 11926068

[B55] Zolkin A. L. Matvienko E. V. Koval Y. N. (2023). “ Use of a new breeding methods for creation of breeds with desired properties and traits”, in: BIO Web of Conferences (Les Ulis, France: EDP Sciences), 67. 01005. doi: 10.1051/bioconf/20236701005

[B56] Żur I. Adamus A. Cegielska-Taras T. Cichorz S. Dubas E. Gajecka M. . (2022). Doubled haploids: Contributions of Poland’s academies in recognizing the mechanism of gametophyte cell reprogramming and their utilization in breeding of agricultural and vegetable species. Acta Soc Bot. Pol. 91, 9128. doi: 10.5586/asbp.9128

